# Cardiopulmonary arrest upon admission caused by pilsicainide hydrochloride intoxication: A case report

**DOI:** 10.1002/ccr3.7448

**Published:** 2023-06-22

**Authors:** Yoshihito Takahashi, Hiroshi Matsuura, Hiroshi Hino, Satoru Chujoh, Masafumi Kishimoto

**Affiliations:** ^1^ Osaka Prefectural Nakakawachi Emergency and Critical Care Center Osaka Japan

**Keywords:** cardiac arrest, hemodialysis, intoxication, percutaneous cardiopulmonary support, pilsicainide hydrochloride

## Abstract

A 22‐year‐old male presented to our hospital after receiving 2450 mg of pilsicainide hydrochloride. Subsequently, he experienced cardiac arrest, and percutaneous cardiopulmonary support was introduced to maintain his circulation. After 3 days of intensive care, he regained consciousness and was transferred to another hospital for treatment related to psychological problems.

## 1 INTRODUCTION

Pilsicainide hydrochloride is included in the class IC antiarrhythmic drug group.^1^ It increases the P‐Q segments, QRS wave, and Q‐Tc segments on electrocardiogram (ECG), and its serum concentration is strongly correlated with the percentage prolongation of the P‐Q segments.^2^ Pilsicainide hydrochloride was developed in Japan and was launched in 1991. It was one of the most commonly prescribed antiarrhythmic drugs in Japan until recently. Here, we report the case of a patient who received a large dose of pilsicainide hydrochloride in a suicidal attempt.

## 2 CASE HISTORY

A 22‐year‐old male patient was transported to our hospital after receiving several drugs. Upon admission, the emergency medical system team reported that he had a Glasgow Coma Scale score of 3, a blood pressure of 153/111 mmHg, a heart rate (HR) of 47 beats/min, and a palpable radial artery. Although the patient was initially assessed to be breathing, subsequent evaluation revealed that he was in fact in cardiac arrest with pulseless electrical activity upon admission. We found that he had taken a high dose of pilsicainide hydrochloride (2450 mg), which was prescribed to his mother. Moreover, he had a medical history of a psychiatric disorder and methamphetamine use.

Upon admission, the patient's height was 180 cm, and his weight was 77.7 kg. Laboratory findings are presented in Table 1.

**TABLE 1:** Laboratory findings upon admission.

**Blood gas analysis (FiO**_**2**_ **: 0.21)**			**Biochemistry**		
PH	7.295		TP	6.5	g/dL
PaO_2_	33.1	mmHg	Alb	4.1	g/dL
PaCO_2_	43.7	mmHg	Na	139	mEq/L
HCO_3_^−^	20.6	mmol/L	K	3.9	mEq/L
BE	−5.5	mmol/L	Cl	101	mEq/L
Anion Gap	11.7		BUN	4.9	mg/dL
Glu	152	mmol/L	Cre	32	IU/L
Lac	6.7	mmol/L	AST	15	IU/L
**Blood count**			ALT	5	IU/L
WBC	10,460	μL	LDH	153	IU/L
Seg	45.6	%	γ‐GT	7	IU/L
Ly	2.6	%	CK	132	IU/L
Eo	2.3	%	CRP	< 0.05	mg/dL
Ba	0.5	%	**Urine drug screen**		
Hb	15.4	g/dL	METH	‐	
Plt	22.2	×10^4^/μL	BAR	‐	
**Coagulation**			BZO	‐	
PT	11.7	s	COC	‐	
PT‐INR	1.03		THC	‐	
APTT	25.3	s	TCA	‐	
Fib	141.7	mg/dL	MOR	‐	

**FIGURE 1:**
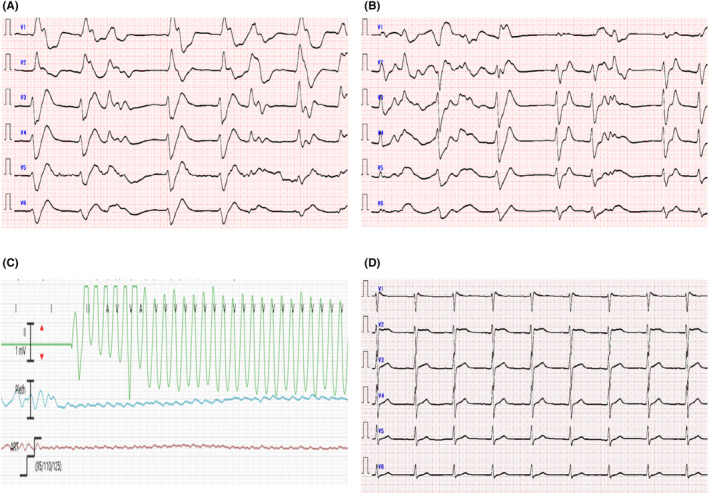
ECG findings. (A) Upon admission. (B) ECG with a wide QRS complex of 210 ms before starting dialysis. (C) VT on the monitor. (D) After HD. ECG, electrocardiogram; HD, hemodialysis; VT, ventricular tachycardia.

The ECG showed bradycardia with a wide QRS wave of 146 ms and a HR of 40 beats/min (Figure 1). The initial serum concentration of pilsicainide hydrochloride was abnormally high at 12.46 μg/mL (therapeutic range: 0.2–0.9 μg/mL).

**FIGURE 2:**
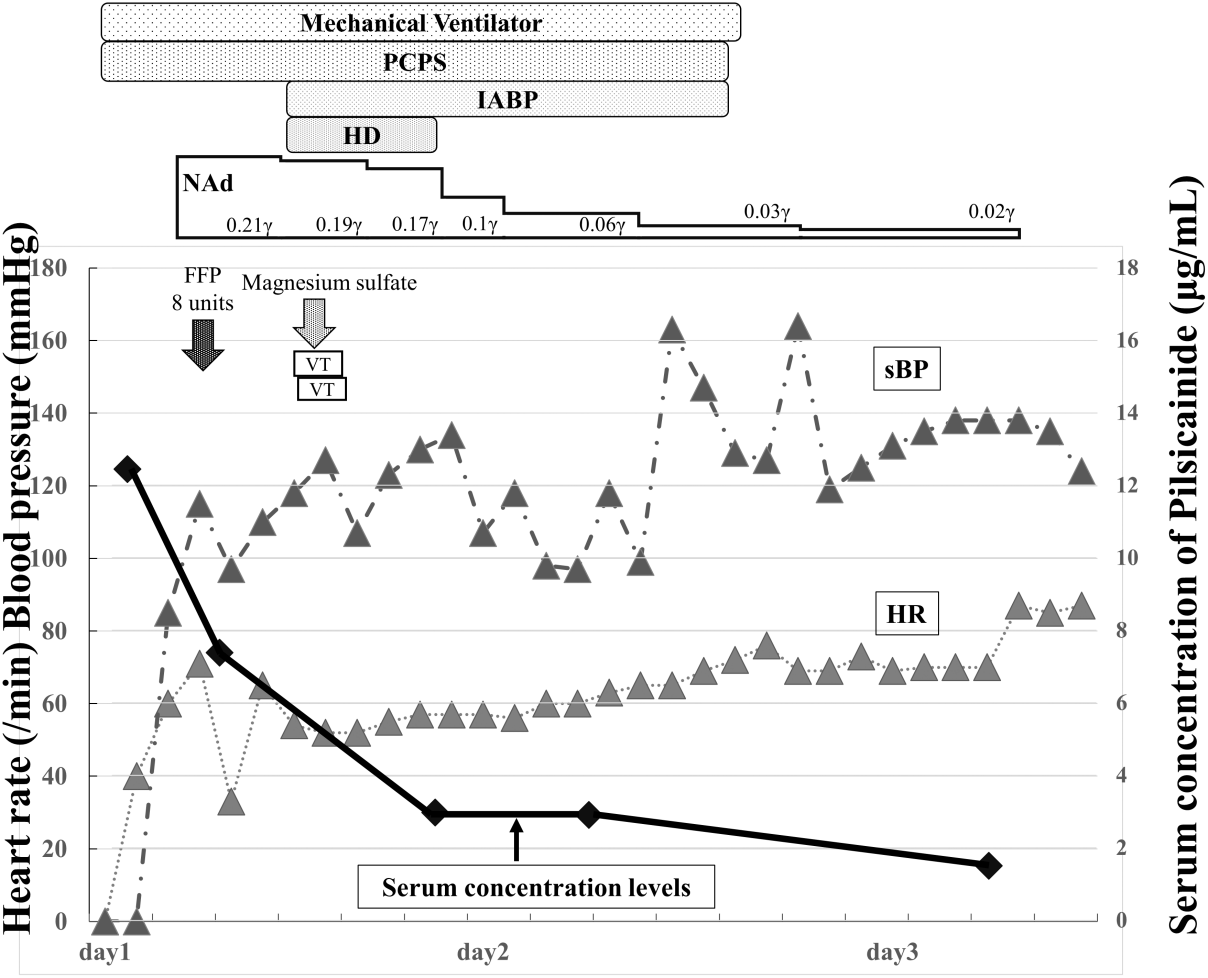
Clinical course of the patient. ECG, electrocardiogram; FFP, fresh frozen plasma; HD, hemodialysis; HR, heart rate; IABP, intra‐aortic balloon pumping; NAd, noradrenaline; PCPS, percutaneous cardiopulmonary support; sBP, systolic blood pressure; VT, ventricular tachycardia.

The patient's clinical course is shown in Figure 2. As the patient was in cardiac arrest upon arrival at our hospital, a peripheral venous catheter was inserted and tracheal intubation was performed. Ten doses of 1 mg of adrenaline were administered for resuscitation; however, spontaneous circulation was not restored. Therefore, percutaneous cardiopulmonary support (PCPS) was established 40 min after arrival using the Getinge CS300 with TERUMO CAPIOX ME‐SP200C to maintain circulation. A contrast‐enhanced computed tomography scan showed no factors affecting respiration and circulation. The patient was admitted to the intensive care unit (ICU), and hemodialysis (HD) was initiated to eliminate the drug using a NIKKISO DBB‐100NX machine. In addition, intra‐aortic balloon pumping (IABP) using a Getinge CS300 was performed to support circulation. Echocardiography in the ICU showed a visual ejection fraction (EF) of approximately 10–15%. After the induction of HD, ventricular tachycardia (VT) was observed (Figure 1). Cardioversion at 200 J using a Stryker LIFEPAK 20e was performed to restore normal sinus rhythm, and magnesium sulfate (20 mEq/L) was administered to prevent arrhythmia. The visual EF on echocardiography gradually improved. No recurrence of arrhythmia was observed, and the serum concentration of pilsicainide hydrochloride had decreased to 2.98 μg/mL 8 h after performing HD. The patient was withdrawn from PCPS and IABP 24 h after arrival at our hospital and was weaned from mechanical ventilation after 28 h. On the third day, the patient's serum concentration of pilsicainide hydrochloride had decreased to 1.51 μg/mL, and he was transferred to another hospital for further treatment related to psychological problems. Upon the transfer to another hospital, his vital signs remained stable, and he experienced no disturbance of consciousness. Five months later, we followed up with the patient and found that his neurological prognosis was favorable and no recurrent arrhythmias occurred.

## 3 DISCUSSION

Pilsicainide hydrochloride is one of the most frequently antiarrhythmic drugs used only in Japan; however, other class IC antiarrhythmic drugs such as flecainide are used worldwide, which may result in similar cases with high mortality rates.^3^ The effective serum concentration range of pilsicainide hydrochloride is 0.2–0.9 μg/mL, with marked P‐Q prolongation reported at levels of 0.98 μg/mL or higher, and adverse effects are likely to occur at levels exceeding 0.9 μg/mL.^4^ The highest serum concentration recorded in our patient was 12.46 μg/mL, and owing to the difficulty in maintaining his circulation upon admission, PCPS was established.

The reported cases of intoxication by pilsicainide hydrochloride overdose are summarized in Table 2.

**TABLE 2:** Reported cases of pilsicainide hydrochloride overdose.

Author (year)	Age/sex	GCS or JCS	BP (mmHg)	Cre (mg/dL)	ECG	Treatment	Dosage (mg)	Maximum serum concentration (μg/mL)
Kanda (1997)	27/M	GCS E3V4M6	60/−	1.30	VT	Gastric lavage, Temporary pacing	750	4.94
Nakajima (2001)	43/M	JCS 20	98/44	Not described	Wide QRS Long QTc	Intubation, IABP, PCPS	2000	4.81
Nakata (2006)	34/M	GCS E4V5M5	74/50	Not described	Wide QRS tachycardia	Intubation, Gastric lavage, Cardioversion, DHP, Magnesium	2500	7.22
Matsuda (2014)	44/M	JCS 1	145/97	1.13	VT	Lidocaine, Temporary pacing	750	5.10
Fujii (2015)	48/M	JCS 1	133/96	1.24	Wide QRS tachycardia	Noradrenaline	4500	7.04
Oshima (2019)	30/M	JCS 1	138/98	0.51	VT	Cardioversion, Magnesium	1000	3.89
Tsuru (2019)	40/M	GCS E1V1M1	–	1.40	Wide QRS long QTc	Intubation, DHP, CHDF	1200	4.49
Our patients (2021)	22/M	GCS E1V1M1	–	1.00	Wide QRS	Intubation, PCPS, IABP, HD, magnesium	2450	12.46

Abbreviations: BP, blood pressure; CHDF, continuous hemodiafiltration; DHP, direct hemoperfusion; HD, hemodialysis; IABP, intra‐aortic balloon pumping; PCPS, percutaneous cardiopulmonary support.

ECG abnormalities such as VT and wide QRS waves were reported. Except for that in an autopsy report,^5^ the serum concentration of pilsicainide hydrochloride on admission was the highest in our patient. A similar case of a patient who experienced a severe shock after receiving 2000 mg of pilsicainide hydrochloride has been reported. The patient's maximum serum concentration increased to 4.81 μg/mL, and he was treated with PCPS and IABP. Moreover, temporary pacing and HD have been reported as common treatments.

Pilsicainide hydrochloride is primarily excreted renally at a high urinary excretion rate of 80% of unchanged drug^6^ and has a blood half‐life of 3.4 ± 0.2 h, which is markedly prolonged to 23.7 ± 0.2 h when creatinine clearance is less than 20 mL/min. There are no known antidotes or antagonists for pilsicainide hydrochloride, and its removal rate is low: 37% on HD, 25% on hemofiltration dialysis, and 45% on plasma exchange. Because a slight decrease in the serum concentration of pilsicainide hydrochloride has been reported to improve symptoms^7^ and because our patient experienced repeated polymorphic VT, we intervened with HD to immediately decrease the serum concentration. Magnesium sulfate was reported^8^ to be another effective treatment option. Therefore, we administered it to prevent arrhythmia in our patient.

## 4 CONCLUSION

We reported a rare case of pilsicainide hydrochloride intoxication due to overdose. Temporary support using PCPS and other multidisciplinary treatments maintained the patient's circulatory status, and he recovered with no impaired consciousness. This report would be useful for treatment planning in similar cases of class IC antiarrhythmic drugs overdose.

## AUTHOR CONTRIBUTIONS

**Yoshihito Takahashi:** Conceptualization; investigation; writing – original draft; writing – review and editing. **Hiroshi Matsuura:** Conceptualization; supervision; writing – original draft; writing – review and editing. **Hiroshi Hino:** Supervision. **Satoru Chujoh:** Supervision. **Masafumi Kishimoto:** Supervision.

## ACKNOWLEDGMENT

We sincerely thank the patient and his family for their support in contributing to this case report.

## CONFLICT OF INTEREST STATEMENT

Authors declare no conflict of interest for this article.

## PATIENT CONSENT STATEMENT

A written informed consent was obtained from the patient and his family for publishing this report according to the journal's patient consent policy.

## Data Availability

The patient's data of this case report are available from the corresponding author on reasonable request.
